# Physical health profile and associated behaviour during the COVID-19 pandemic in patients with bipolar disorder

**DOI:** 10.1192/j.eurpsy.2022.1023

**Published:** 2022-09-01

**Authors:** J.D. Sperling, N. Dalkner, C. Berndt, E. Fleischmann, M. Ratzenhofer, J. Martini, A. Pfennig, M. Bauer, E. Reininghaus, M. Vinberg

**Affiliations:** 1 Psychiatric Center of North Zealand, Psychiatric Research Unit, Hillerød, Denmark; 2 Medical University Graz, Psychiatry And Psychotherapeutic Medicine, Graz, Austria; 3 Faculty of medicine, Carl Gustav Carus University Hospital, Technische Universität Dresden, Department Of Psychiatry & Psychotherapy, dresden, Germany; 4 Mental Health Services in the Capital Region of Denmark, Mental Health Centre North Zealand, Hilleroed, Denmark

**Keywords:** physical health, bipolar disorder, Behavioural changes, Covid-19 pandemic

## Abstract

**Introduction:**

The COVID-19 pandemic has led to an increased psychological strain on public mental health and may impact behavioural, mental, and physical health, presumably with effects on patients with severe mental disorders.

**Objectives:**

This study examines pandemic-related physical and mental health and (compensatory) behavioural changes, in patients with BD as compared to healthy control individuals.

**Methods:**

Physical and mental health and self-reported changes in daily structure and behaviour due to pandemic were assessed using a self-constructed questionnaire and the brief symptom inventory (BSI) from outpatient clinics in Germany, Austria, and Denmark in individuals with BD and a healthy control group.

**Results:**

The present study included 118 individuals with BD and 215 healthy controls. Individuals with BD reported statistically significant higher physical risk burden, increased weight gain, more physical comorbidities, and a decrease in physical activity and they further reported higher rate of COVID-19 testing, had more worries concerning health and experienced more anxiety but less social distancing.

**Conclusions:**

The COVID-19 pandemic seems to have a greater impact on physical health in individuals with BD than in healthy controls. Individuals with BD appear to be having more difficulties compensating their behaviour due to the pandemic which could amplify the effect of risk factors associated with poorer physical health. This highlights the necessity for optimising and targeting the overall treatment of both mental and physical health in patients with BD during periods with far-reaching changes such as COVID-19 pandemic.

**Disclosure:**

No significant relationships.

